# FACT inhibitor CBL0137, administered in an optimized schedule, potentiates radiation therapy for glioblastoma by suppressing DNA damage repair

**DOI:** 10.21203/rs.3.rs-4830689/v1

**Published:** 2024-09-09

**Authors:** Tara A. Barone, Denisha L. Robinson, Jingxin Qiu, Katerina V. Gurova, Andrei A. Purmal, Andrei V. Gudkov, Robert J. Plunkett

**Affiliations:** Roswell Park Comprehensive Cancer Center; Roswell Park Comprehensive Cancer Center; Roswell Park Comprehensive Cancer Center; Roswell Park Comprehensive Cancer Center; Incuron, LLC; Roswell Park Comprehensive Cancer Center; Roswell Park Comprehensive Cancer Center

**Keywords:** Glioblastoma, CBL0137, radiation, ATRX, DNA damage repair

## Abstract

**Purpose:**

Standard-of-care for glioblastoma remains surgical debulking followed by temozolomide and radiation. However, many tumors become radio-resistant while radiation damages surrounding brain tissue. Novel therapies are needed to increase the effectiveness of radiation and reduce the required radiation dose. Drug candidate CBL0137 is efficacious against glioblastoma by inhibiting histone chaperone FACT, known to be involved in DNA damage repair. We investigated the combination of CBL0137 and radiation on glioblastoma.

**Methods:**

*In vitro*, we combined CBL0137 with radiation on U87MG and A1207 glioblastoma cells using the clonogenic assay to evaluate the response to several treatment regimens, and the Fast Halo Assay to examine DNA repair. *In vivo*, we used the optimum combination treatment regimen to evaluate the response of orthotopic tumors in nude mice.

**Results:**

*In vitro*, the combination of CBL0137 and radiation is superior to either alone and administering CBL0137 two hours prior to radiation, having the drug present during and for a prolonged period post-radiation, is an optimal schedule. CBL0137 inhibits DNA damage repair following radiation and affects the subcellular distribution of histone chaperone ATRX, a molecule involved in DNA repair. *In vivo*, one dose of CBL0137 is efficacious and the combination of CBL0137 with radiation increases median survival over either monotherapy.

**Conclusions:**

CBL0137 is most effective with radiation for glioblastoma when present at the time of radiation, immediately after and for a prolonged period post-radiation, by inhibiting DNA repair caused by radiation. The combination leads to increased survival making it attractive as a dual therapy.

## Introduction

Glioblastoma is the most frequently occurring brain tumor and is highly aggressive with a 5-year survival rate of only 6.9% [1]. Standard-of-care is surgical debulking, followed by temozolomide and radiation. Median survival with treatment is only around 15 months and tumors can be resistant to radiation, regularly recurring after chemoradiation [2, 3]. Additionally, radiation affects neurocognition [4]. New combination therapies which increase efficacy of radiation, possibly reducing the dose required, are needed.

In previous studies, we and others demonstrated that histone chaperone FACT (Facilitates Chromatin Transcription) inhibitor CBL0137 was efficacious in preclinical models of glioblastoma [5, 6]. We established the FACT subunits, SSRP1 and SPT16, are upregulated in GBM [5]. CBL0137 traps FACT in chromatin, thereby inactivating it, which causes inhibition of NF-κB-mediated transcription and activation of p53, making it an excellent anti-cancer candidate [7]. The drug has also been shown to sensitize cancer cells, including glioblastoma stem cells and medulloblastoma, to radiation [8, 9]. Since FACT is implicated in DNA repair and locates to areas of double-strand DNA breaks [10], which radiation causes, its downregulation would then suppress DNA repair.

In this study, we demonstrate the combination of CBL0137 and radiation is superior to either alone *in vitro*. We establish that giving CBL0137 two hours prior to radiation and having the drug present during and for a prolonged period post radiation, is an optimal schedule for combination treatment *in vitro*. We also demonstrate that the drug inhibits DNA damage repair (DDR) following radiation in GBM cells. In addition to its effects on DNA repair through FACT, we show CBL0137 affects the subcellular distribution of histone chaperone ATRX, a molecule which is also involved in DNA repair. *In vivo*, just one dose of CBL0137 is efficacious and the combination of CBL0137 with radiation increases median survival over either monotherapy. These results suggest that CBL0137 inhibits growth of GBM and sensitizes it to radiation. Thus, the combination of CBL0137 with radiation treatment deserves further evaluation.

## Materials and Methods

### Cell Culture

A1207 (a gift from Stuart Aaronson, Mt. Sinai Hospital, New York, NY; RRID:CVCL 8481) and U87MG (American Tissue Type Collection; RRID:CVCL 0022) were cultured as outlined in [5].

### Drug Preparation

CBL0137 was provided by Incuron and dissolved at 20mM stock solution in dimethyl sulfoxide (DMSO) for *in vitro* experiments. Stock was diluted to 0.2mM in serum free media before final dilution in culture plates. For *in vivo* experiments, CBL0137 was prepared at 10 mg/mL in 5% dextrose.

### Clonogenic Assay

**Figure 1:**
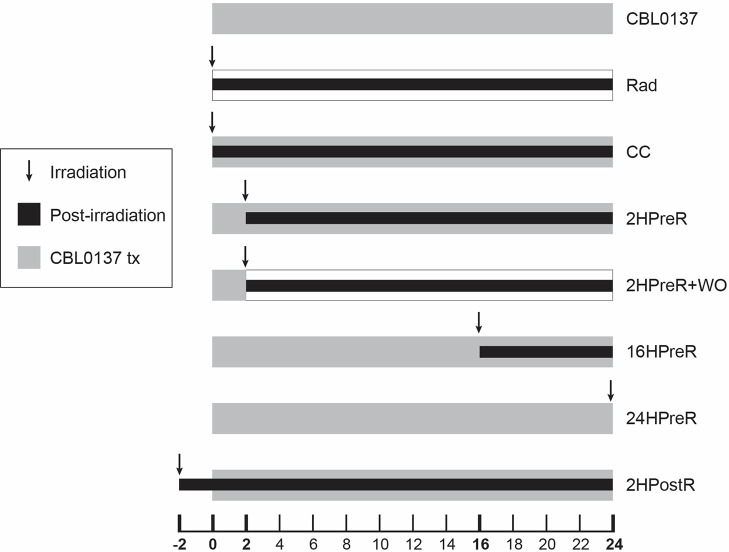
Graphic of clonogenic assay treatment regimens. U87MG or A1207 GBM cells were irradiated with 2 or 4Gy alone or with CBL0137 at 0.2μM or 0.4 μM in the following combination treatment regimens: CBL0137 = drug alone; Rad = radiation alone; CBL0137 added: CC = concurrently; 2HPreR = 2 hours before radiation; 2HPreR+WO = 2 hours before radiation and then washed out; 16HPreR = 16 hours before radiation; 24HPreR = 24 hours before radiation, 2HPostR = 2 hours after radiation. Cells were exposed to CBL0137 for a total of 24 hours except in the case of 2HPre+WO

Our assay was based on Franken et al. [11]. A1207 (300 cells/well) or U87MG (400 cells/well) were plated in a 6-well plate and allowed to attach for 2 hours. CBL0137 was then added to wells at a final concentration of either 0.2μM or 0.4μM. Control wells received DMSO diluted in media. For all groups, CBL0137 media was removed and replaced with fresh media 24 hours after treatment initiation except for 2-hour pre-radiation + washout (2HPreR + WO). In that group, cells were treated with CBL0137 for two hours before the media was removed, the wells washed with DPBS (Gibco/ThermoFisher Scientific) and fresh media was added. Cells were treated with CBL0137, and then concurrent with or at 2, 16, or 24 hours after CBL0137 treatment, they were irradiated at 2, 4, or 6 Gy in a JL Shepard Mark I irradiator. For the 2-hour post group (2HPostR), cells were treated with CBL0137 2 hours post-radiation. Treatment is summarized in Fig. 1. Treatments were administered in triplicate. Eleven (A1207) or 12 (U87MG) days after plating, colonies were stained and counted as detailed in Supplementary Methods. Conventionally, clonogenic results are graphed as survival curves and the curves are compared. However, since we were comparing several different treatment regimens, we opted to use bar graphs to display the granularity of the data and compare individual data points among several regimens using ANOVA (GraphPad Prism 9.5.1).

### Fast Halo Assay (FHA)

We assayed for DNA double-strand breaks using the non-denaturing alkaline FHA according to Sestilli et al. [12]. Briefly, A1207 or U87MG cells were plated in 6-well plates at 200,000 or 250,000 cells/well, respectively, and allowed to sit overnight at 37°C in a 5% CO2 incubator. Cells were treated with CBL0137 two hours before irradiation. CBL0137 and radiation dose along with cell harvesting, application to slides, and processing are detailed in Supplementary Methods. Diffusion of DNA fragments (halos) was measured, using Halo J [13], on at least 50 cells per slide in triplicate experiments. The nuclear diffusion factor (NDF, (Halo area – Halo nucleus area)/Halo nucleus area) was calculated for each cell using Halo J and the mean and SE for each treatment was graphed and compared using one-way ANOVA (GraphPad Prism 9.5.1)

### Glioblastoma Immunohistochemistry Panel

Cultures of A1207 or U87MG cells were harvested, pelleted, fixed, paraffin embedded, and stained for p53, Ki67, ATRX, IDH1 and GFAP as described in Supplementary Materials and Methods.

### ATRX Western Blots

Cells were treated with a dose-range of CBL0137 (0.2–3.2μM) for 1 hour, then harvested, lysed and proteins separated by Western blot. Blots were probed with antibodies to ATRX, SSRP1 and loading control as described in Supplementary Materials and Methods.

### Orthotopic Models and Survival Studies

All *in vivo* studies were reviewed and approved by the Roswell Park Comprehensive Cancer Center Institutional Animal Care and Use Committee and adhere to the Guide for the Care and Use of Laboratory Animals. Eight to nine-week-old male athymic nude mice (Taconic RRID:IMSR TAC:NCRNU and Envigo RRID:IMSR ENV:HSD-069) were implanted orthotopically with 5 × 10^5^ of either U87MG or A1207 human glioblastoma cells according to Barone et al. [5]. Animals were divided into 4 groups/cell line: Control, CBL0137 alone, radiation alone, or combination CBL0137 + radiation (Combo). One week after U87MG and two weeks after A1207 tumor inoculation, reflecting the tumors’ different growth rates, animals were anesthetized with isoflurane, and CBL0137 or 5% dextrose vehicle was administered retro-orbitally at 80mg/kg, 2 hours before irradiation. For irradiation, anesthesia was induced with isoflurane and maintained with a nose cone throughout the radiation procedure. Each mouse was laid on its stomach and the neck and body shielded with lead, leaving the head exposed. A 1 × 2 cm cone with Thoraeus filter was advanced to the top of the skull and the brain was irradiated with 8 Gy using a Philips RT 250 Orthovoltage X-Ray unit. Sham irradiation was administered to mice receiving CBL0137 alone. Mice were euthanized when they showed signs of tumor burden such as anorexia, hunched posture and lack of ambulation. Survival data were plotted on a Kaplan-Meier curve and analyzed using the Mantel-Cox log-rank test (GraphPad Prism 9.5.1).

## Results

### Combination of CBL0137 and Radiation Inhibits GBM Cell Colony Growth Better Than Either Alone and Schedule Matters

**Figure 2:**
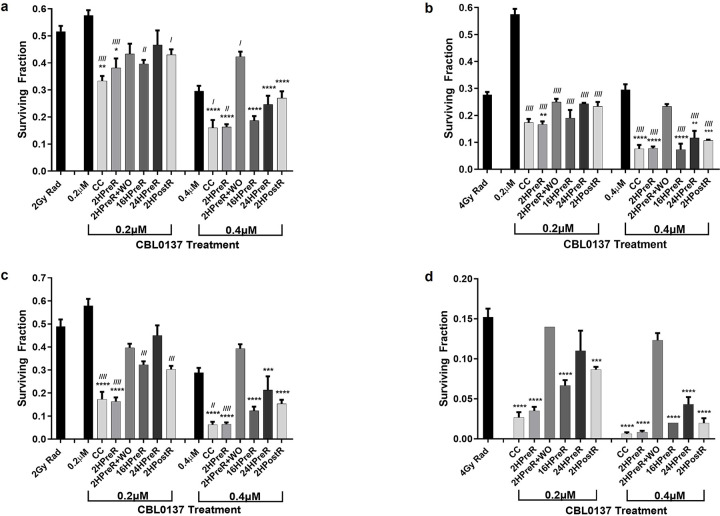
Effect of several different CBL0137 and radiation treatment regimens, outlined in Fig. 1, on U87MG (**a, b**) and A1207 (**c, d**) GBM cells as assessed using the clonogenic assay. Cells were irradiated with 2 Gy (**a, c**) or 4Gy (**b, d**) or treated with CBL0137, at 0.2μM or 0.4 μM, alone or in the following combination treatment regimens: Rad = radiation alone; CBL0137 added: CC = concurrently; 2HPreR = 2 hours before radiation; 2HPreR+WO = 2 hours before radiation and then washed out; 16HPreR = 16 hours before radiation; 24HPreR = 24 hours before radiation, 2HPostR = 2 hours after radiation. Cells were exposed to CBL0137 for a total of 24 hours except in the case of 2HPre+WO. In graph d, the CBL0137 alone group (SF=0.29) is not shown as it causes the y-axis scale to be too large to view the smaller value bars and all groups are significantly different from drug alone. Groups were compared by one-way ANOVA. *, / symbolizes the group as compared with radiation or CBL0137 alone, respectively. * or ^/^ p≤ 0.05, ** or ^//^ p≤0.01, *** or ^///^ p≤0.001, **** or ^////^ p≤0.0001

In studies to determine the effect of CBL0137 combined with radiation (Fig. 1), colony forming assays revealed 2-hour pretreatment with CBL0137 (2HPreR) before radiation was always significantly better than either drug or radiation alone, at reducing the surviving fraction of cells, in both U87MG and A1207 cell lines (Fig. 2). Giving dual treatment concurrently (CC) was also significantly better than either alone, with one exception (Fig. 2b). We excluded data from the 6Gy radiation dose, since the mean surviving fraction was very low, with only a few countable colonies in most wells, making the data unreliable since it was subject to considerable variation from too few colonies. Based on our results, for all subsequent *in vitro* and *in vivo* studies, we used the 2-hour CBL0137 pretreatment regimen.

### Duration of Exposure to CBL0137 After Radiation Leads to Significantly Different Cell Survival

Replicating the optimal schedule of a 2-hour CBL0137 pretreatment before radiation, but washing the drug away before radiation (2HPreR + WO), allowing no time with CBL0137 after radiation, was never significantly better than radiation alone and at 2Gy, was not better than drug alone either, in both cell lines (Fig. 2a,c). In fact, leaving the drug with cells after 2-hour CBL0137 pretreatment before radiation was always significantly better than washing drug away for A1207 and better for U87 with the higher dose of drug. The significant difference between washing drug away after 2 hours and leaving drug with the cells in both the concurrent and 2-hour pretreatment regimens was particularly noticeable at the 0.4 μM dose of drug. Also notably, at the 2Gy radiation dose, pretreatment for 24 hours with 0.2μM CBL0137, but removing drug before radiation (24HPreR), did not lead to significantly different cell survival from either monotherapy. However, at this radiation and drug dose, 16-hour pretreatment (16HPreR) giving 8 hours of post-radiation exposure, led to a significant difference over drug therapy alone, (Fig. 2a, c). We see this difference with the 2HPostIR group (24 hours drug exposure) as well.

### A1207 and U87MG Have Different Sensitivities to Treatment Regimens and ATRX May Be Involved

**Figure 3:**
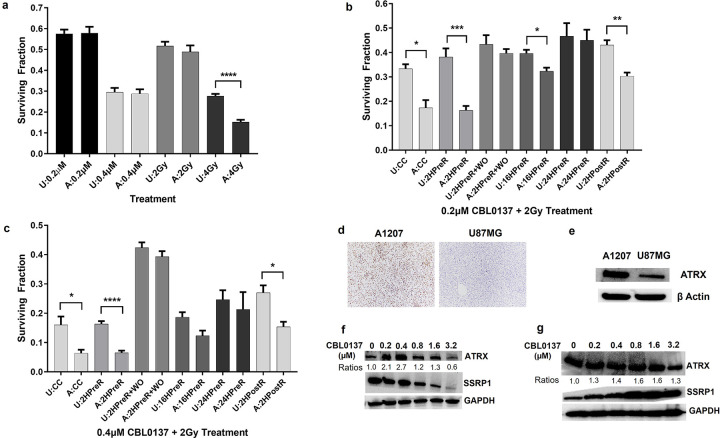
*In vitro* comparison between U87MG (U) and A1207 (A) treated with combinations of radiation and CBL0137. Cells were irradiated or treated with CBL0137 alone (**a**) or a combination of 2Gy with CBL0137 at 0.2 μM (**b**) or 0.4 μM (**c**) and assessed using the clonogenic assay. Data is taken from Fig.2. U87MG and A1207 immunohistochemical staining (**d**) and Western blots (**e**) for ATRX. Effect of a range of CBL0137 (0 – 3.2μM) concentrations on ATRX expression in the soluble fraction (cytoplasm+nucleoplasm) (**f**) and insoluble fraction (chromatin) (**g**) of A1207 after 1 hour, shown by Western blot. Quantified band intensities were normalized to GAPDH and ATRX ratios computed normalized to control. Combination treatment regimens (CBL0137 added): CC = concurrently; 2HPreR = 2 hours before radiation; 2HPreR+WO = 2 hours before radiation and then washed out; 16HPreR = 16 hours before radiation; 24HPreR = 24 hours before radiation, 2HPostR = 2 hours after radiation. Cells were exposed to CBL0137 for a total of 24 hours except in the case of 2HPre+WO. Clonogenic assay groups were compared using student t-test. *p≤ 0.05, **p≤0.01, ***p≤0.001, **** p≤0.0001

In vitro, A1207 and U87MG were similarly affected by 2 Gy radiation and either dose of CBL0137 monotherapy (Fig. 3a). In fact, the relative IC50 for both was 0.254 μM by clonogenic assay. However, A1207 was significantly more sensitive to 4Gy (Fig. 3a) and this sensitivity even prompted lowering the range of the y-axis and excluding the drug treatment group alone in Fig. 2d, as all treatments were significantly different from drug alone. While we established that dual treatment of each cell line is significantly different from either monotherapy when given concurrently or in a 2-hour pretreatment regimen at 2Gy, there is also a significant difference *between* cell lines when given in these regimens (Fig. 3b & c). To profile any differences between the cell lines that may contribute to the sensitivity differential, we performed a standard GBM immunohistochemical clinical panel on A1207 and U87MG. Pathologist review deemed both, p53 mutant, Ki67 positive, GFAP negative and IDH1 wild-type. However, almost all A1207 cells stained ATRX positive while only a few U87MG did, leading A1207 to be labelled ATRX wild-type and U87MG as mutant (Fig. 3d). U87MG produces half the amount of ATRX upon protein blotting (Fig. 3e). Since ATRX is a histone chaperone much like FACT, we sought to determine if CBL0137 affects cellular ATRX. We probed for FACT subunit SSRP1 as a comparison since CBL0137’s effects on FACT are known and detailed in [7]. In A1207, SSRP1 shows the expected protein decrease in the soluble fraction (cytoplasm + nucleoplasm) at and above 0.8μM and increase in pellet fraction (chromatin) with increasing CBL0137 dose (Fig. 3f&g). ATRX more than doubles in the soluble fraction with addition of 0.2 and 0.4μM CBL0137 before returning close to basal levels at and above 0.8μM (Fig. 3f). ATRX increases over 1.5x in the chromatin fraction (Fig. 3g). Soluble ATRX in U87MG was undetectable and chromatin levels were not high enough to document a discernable difference with increasing doses of CBL0137 and are not shown.

### CBL0137 Inhibits DDR After Radiation

**Figure 4:**
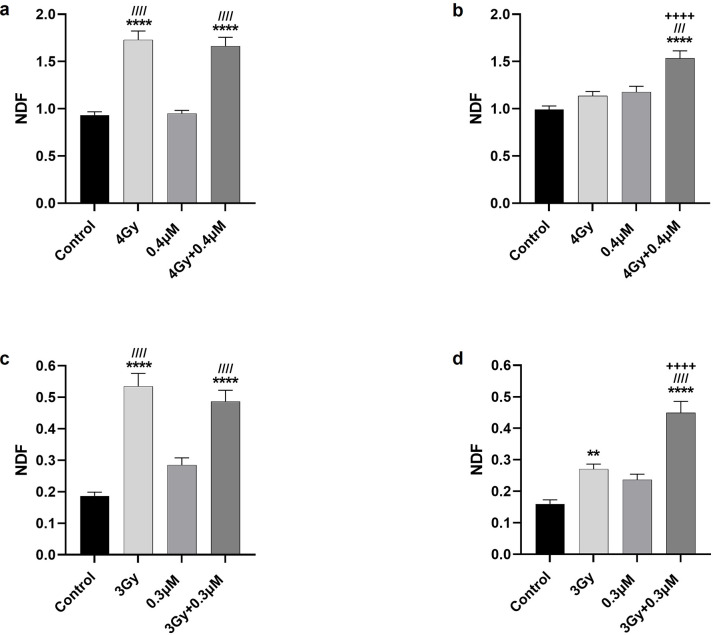
Double-strand break DNA damage to U87MG (a, b) and A1207 (c, d) cells assessed using the fast HALO assay. Cells were treated with 0.4 or 0.3 μM CBL0137, 2 hours before irradiation with 4 or 3 Gy, respectively. Cells were harvested immediately (a, c) or 22 hours after radiation (b, d) and the fast Halo assay was performed. The nuclear diffusion factor (NDF) was graphed and compared using one-way ANOVA. *, /, + symbolizes the group as compared to control, CBL0137, and radiation, respectively. **p≤0.01, ^///^p≤0.001, ****, ^////^ or ^++++^ p≤0.0001

Since both FACT and ATRX are implicated in DDR inhibition, we assessed DNA double-strand breaks (DSB) as a measure of DNA damage after radiation and/or CBL0137. Typically, γH2AX is assayed to measure these breaks since it is an early molecule involved in DNA repair of DSB [14]. However, FACT is intimately involved in the formation of γH2AX [10], making γH2AX an unreliable measure of DSB in response to CBL0137. Therefore, we chose the Fast Halo Assay (FHA) to assess DNA fragment diffusion from the nucleus after DSB damage [12]. We treated cells with CBL0137 two hours before radiation and harvested cells immediately (Fig. 4a&c) or 22 hours (Fig. 4b&d) after radiation and quantified the nuclear diffusion factor (NDF). Immediately after radiation, U87MG cells showed a significant increase in NDF (p < 0.0001) while cells treated with CBL0137 alone showed no difference from control. Cells treated with both drug and radiation showed the anticipated significant increase (p < 0.0001, Fig. 4a). Twenty-two hours after radiation, monotherapies were not significantly different from control. However, the dual treatment group remained significantly different from control (p < 0.0001) and from either treatment alone (Fig. 4b). Results for A1207 were similar, especially immediately after radiation (Fig. 4c). Twenty-two hours after radiation, the irradiated cells, while showing an almost 50% decreased NDF, were still statistically different from control (p < 0.01) which was not surprising given that A1207 is more sensitive to radiation than U87MG. Importantly, the combination group was highly different (p < 0.0001) than control and from either treatment alone (p < 0.0001, Fig. 4d).

### CBL0137 with Radiation Increases Survival in an Orthotopic GBM Mode

**Figure 5:**
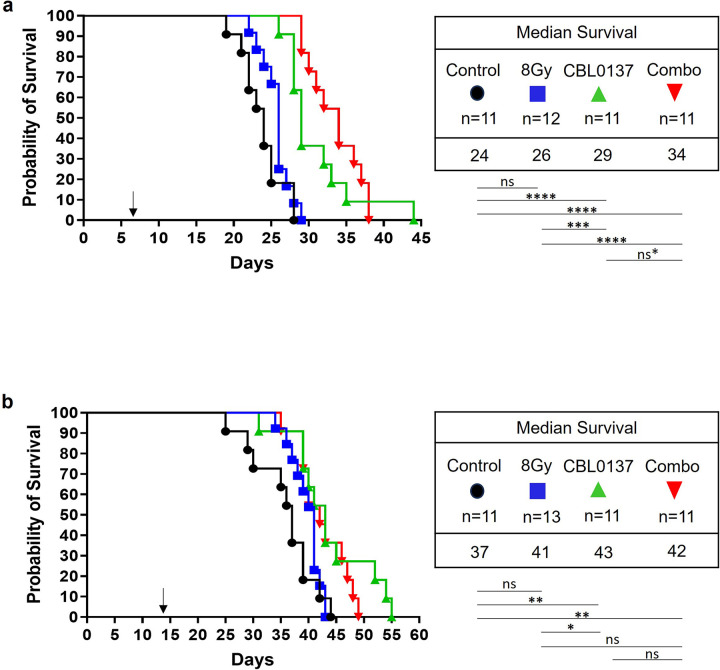
Effect of 8Gy radiation or CBL0137 alone or in combination, on the survival of mice bearing U87MG (**a**) or A1207 (**b**) orthotopic GBM tumors. Mice were given CBL0137 or vehicle retro-orbitally at 80 mg/kg, 2 hours before their heads were irradiated or sham irradiated with 8Gy. Treatment was administered 7 days after U87MG or 14 days after A1207 tumor inoculation (denoted by arrow). Survival curves were compared with the Mantel-Cox log-rank test. *p≤ 0.05, **p≤0.01, ***p≤0.001, **** p≤0.0001 * If the one longer surviving CBL0137-treated animal is removed from the data, the comparison of CBL0137 and Combo is highly significant (p<0.01)

In the orthotopic U87MG model, 80mg/kg CBL0137 given only once at 7 days post inoculation, significantly increased survival over controls (p < 0.0001). However, radiation alone did not. The combination increased survival significantly over control (p < 0.0001), radiation (p < .0001) but not over CBL0137 alone, despite a 5 day increase in median survival and the separation in curves (Fig. 5a). For the orthotopic A1207 model, CBL0137 alone given at 14 days post inoculation, increased survival significantly over controls (p < 0.01), while radiation alone did not. Combination treatment was significantly better than control (p < 0.01) only (Fig. 5b).

## Discussion

Radiation, which is standard for glioblastoma patients, induces lethal DSBs in DNA. Rapid repair of these DNA breaks leads to radio-resistance [15]. Studies have shown that impairing DSB repair overcomes this radio-resistance in GBM [16].

CBL0137 is a small molecule drug which activates p53 while inhibiting the NF-kB pathway without genotoxicity [7]. It demonstrates efficacy against several cancers in preclinical studies [5, 17, 18] and is currently in clinical trials. We established this drug to be effective at increasing survival in models of temozolomide-sensitive and -resistant GBM and others showed it to be effective against GBM stem cells [5, 6]. Given that investigations implicate NF-kB in the radio-resistance of neoplasms [19, 20] and CBL0137 has been shown to enhance the efficacy of radiotherapy [8, 9], we chose to combine CBL0137 and radiation in our studies on GBM, to expand on our previous work [5].

Our data shows that the timing of drug administration is critical for efficacy. *In vitro*, 2-hour pretreatment with CBL0137 was always significantly different than either treatment alone. Concurrent treatment was also significantly effective with only one exception. Using the 2-hour pretreatment regimen, but washing the drug away before radiation, (2HPreR + WO) reveals the 2-hour pretreatment, by itself, does not alter the cells to be more sensitive to radiation, since this group was not significantly different from either monotherapy. This becomes quite clear at 4Gy radiation with higher dose of drug, as the treatment leaves cells no different than those treated with radiation alone. Additionally, 24-hour pretreatment was also not different from either monotherapy at the low dose of radiation. Both treatment schedules allow no time with CBL0137 after radiation, while concurrent and 2-hour pretreatment allow for drug to be in contact with newly irradiated cells the longest (24 and 22 hours respectively). However, this does not tell the complete story, as exposure to CBL0137 for 22 hours, but applied 2 hours *after* radiation, did not lead to significant differences from either monotherapy at 2Gy radiation. This led us to understand the importance for CBL0137 to be present at the time of radiation, in the immediate post-radiation period, and for an extended time following radiation. Immediately after radiation, histone chaperone FACT locates to areas of damage, depositing H2AX which potentiates DNA damage signaling, initiating the DNA repair cascade [21,22]. Other studies have shown that inhibiting DNA repair potentiates radiation and DNA damage response inhibitors are currently in clinical trials for glioma [8, 9, 23]. Indeed, our FHA results show that CBL0137 inhibits repair of DSBs caused by radiation. CBL0137 monotherapy did not cause significant DNA damage by itself, as in the original literature [7]. However, in our study, 24 hours after CBL0137 administration to A1207, the CBL0137 alone group does show more damage than control, bordering on significance (p < 0.06). This result aligns with other studies in which DNA damage occurs following CBL0137 administration [9, 24]. Studies show that most DSB repair is finished within a few hours but can take over 24 [25]. Applying CBL0137 two hours post-radiation (2HPostR) likely allowed for immediate repair to begin since this group was not different from radiation alone. Exposure to CBL0137 for a shortened time post-radiation, such as the 8 hours offered in the 16-hour pretreatment group, led to significant differences from one but not both therapies at the 2Gy radiation level. Despite only being different from one therapy, this treatment was close to significance for both. Perhaps drug presence at the time of radiation delayed immediate repair, but late repair could have occurred. Under normal circumstances, FACT aids in promoting transcription after damage is repaired [21]. At the higher dose of drug, it’s difficult to delineate the contribution of transcription inhibition versus DDR inhibition. These results informed our subsequent studies and are meaningful when choosing a potential regimen for patients. The drug must be present during, immediately after, and for up to 24 hours following radiation.

Despite A1207 and U87 having the same relative IC50 for CBL0137, A1207 was more sensitive to the higher radiation dose. Additionally, A1207 was more sensitive to combination treatment at 2Gy with longer CBL0137 exposure times, leading us to start an investigation into the differences between the cell lines. In the limited survey of GBM markers, very few U87MG cells stained positive for ATRX while almost all cells were positive in the A1207 sample. This result is consistent with a search of the Gene Expression and Mutation in Cancer Cell Line database which combines searches of the CCLE, COSMIC, and NCI60 databases [26]. Both the COSMIC and CCLE databases show that the U87MG cell line harbors a missense mutation in ATRX, causing a reduction in expression [26]. A1207 has no ATRX mutation listed in the Cellosaurus database [27]. ATRX, like FACT, is a histone chaperone which is involved in transcription and response to DNA damage [28, 29]. In glioma, loss of ATRX results in genomic instability and impaired NHEJ repair of double-strand breaks, making it more responsive to DNA damaging therapies such as radiation [28, 30]. In addition, ATRX protein associates with both subunits of FACT [curated in 31]. Our results show that CBL0137 is affecting ATRX levels in the subcellular cytosolic and nuclear compartments. CBL0137 could render both ATRX and FACT less effective, allowing a double-hit to the DNA damage response and transcription machinery. Owing to the significantly lower expression of ATRX in U87MG, cells may not be as dependent on ATRX for DNA repair so that CBL0137 may primarily only affect the FACT DNA repair path. Our results and the fact that ATRX mutations exist in a majority of gliomas [32], certainly make ATRX an interesting molecule for future study with CBL0137 administration. It’s also unclear if there’s any interplay between CBL0137 and MGMT which would make A1207 more sensitive to the combination treatment. In our previous study [5], we found A1207, but not U87MG, expresses MGMT, which renders Temozolomide, ineffective. The phenotypic differences we see between our cell lines most likely reflects GBM diversity among patients.

*In vivo*, a single dose of CBL0137 was effective at increasing survival significantly over control for both cell lines, as seen in our previous study [5]. Radiation increased survival by only a few days for each cell line, which was not significantly different from control, a result different from our *in vitro* results, leading us to conclude these cell lines may be resistant, *in vivo*, to the high dose of radiation. This difference between *in vitro* and *in vivo* resistance has been seen in GBM [33]. Given our robust in vitro combination treatment results, we sought to test this dual treatment *in vivo*. In the U87MG model, despite the five-day increase in median survival, the combination treatment did not reach significance over drug alone. The lone long-term survivor in the drug monotherapy group negated the trend apparent in the curve separation. Surprisingly, for A1207, combination treatment was not better than either monotherapy even though *in vitro*, A1207 cells are more sensitive to combination treatment. Most likely, this was due to initiating treatment at a suboptimal time in tumor progression. We chose the 14-day time point to be sure established tumor was present based on growth characteristics and reflecting a treatment time point from our previous study [5]. However, there must be other confounding factors at play such as microenvironment protein interactions or changes to proteins within the tumor once the cells are growing in the brain environment [33]. Additionally, we chose to give only one combination dose, which is different from the fractionated dosing protocol in Tallman et al. [9]. A single dose of CBL0137, given to mice with large tumors, was effective in our previous study [5], and we wanted to avoid the confounding influence of multiple doses of anesthesia. Isoflurane has been shown to enhance the malignant potential of glioblastoma stem cells *in vitro* and *in vivo* through enhancing migration [34]. Sevoflurane promotes the invasion and migration of U87 specifically [35].

This study demonstrates the radio-sensitizing properties of CBL0137 in GBM and establishes the need for CBL0137 to be present at the time of radiation with extended drug exposure post-radiation, which is clinically achievable as CBL0137 has a half-life of over 24 hours in the human body [36]. It also validates our previous observation that CBL0137 is efficacious for GBM, with a single dose, delaying tumor progression, thereby increasing survival. These results show CBL0137 therapy may provide clinical benefit not only by slowing tumor progression, but also enhancing the effect of radiation treatment, possibly reducing the dose required.

## Data Availability

The datasets analyzed during the current study are available from the corresponding author on reasonable request.
